# Another Medical College in Philadelphia

**Published:** 1846-04

**Authors:** 


ARTICLE IX.

### ANOTHER MEDICAL COLLEGE IN PHILADELPHIA.

An Institution with the name of "Franklin Medical College" has been chartered by the Legislature of Pennsylvania, to be located in Philadelphia, making the fourth Medical School in that city. "It is obvious," says the announcement, "that there must be limits to the size of classes that can be usefully instructed in the demonstrative branches of medicine, and those who are acquainted with the Medical Schools of Philadelphia, will recognise the propriety of an extension of the facilities now offered."

We have frequently thought that medical men, who so often complain of the want of respect shown to the profession, should be careful themselves not to set the example. Have there been no physicians in America deserving the honor, that Philadelphia should adopt for her Medical Colleges the names of Jefferson and Franklin, already sufficiently honored in this respect? Certainly Wistar and many other of her sons deserve well to be remembered when new Medical Colleges are to be christened. The following are the names of the faculty of the new Institution. D. B.

PAUL BECK GODDARD, M. D., *Anatomy and Histology.*

C. C. VAN WYCK, M. D., *Principles and Practice of Surgery.*

MEREDITH CLYMER, M. D., *Principles and Practice of Medicine.*

JOHN BARCLAY BIDDLE, M. D., *Materia Medica aud Therapeutics.*

DAVID HUNTER TUCKER, M. D., *Obstetrics and Diseases of Women and Children.*

LEVIN S. JOYNES, M. D., *Physiology and Legal Medicine.*

JAMES B. ROGERS, M. D., *General and Organic Chemistry.*

JOHN BARCLAY BIDDLE, M. D., *Dean of the Faculty.*

JOSEPR LEIDY, M. D., *Demonstrator of Anatomy.*

